# A Rare Case of Chronic Myelomonocytic Leukemia in a Patient With Sarcoidosis: Diagnostic and Immunologic Challenges

**DOI:** 10.7759/cureus.83978

**Published:** 2025-05-12

**Authors:** Ani Toloraia, Dimitri Daraselia, Ninaka Gvamberia, Marika Todua

**Affiliations:** 1 Internal Medicine, Tbilisi State Medical University, Tbilisi, GEO; 2 Internal Medicine, Christliches Krankenhaus Quakenbrück (CKQ), Quakenbrück, DEU

**Keywords:** bone marrow biopsy, chronic myelomonocytic leukemia (cmml), clonal hematopoiesis, cytopenia, diagnostic challenge, extrapulmonary sarcoidosis, immune dysregulation, myelodysplastic/myeloproliferative neoplasm, sarcoidosis, soluble interleukin-2 receptor (sil-2r)

## Abstract

The coexistence of sarcoidosis and chronic myelomonocytic leukemia (CMML) is exceedingly rare and poses significant diagnostic challenges due to overlapping clinical, radiologic, and immunologic features. Sarcoidosis, a systemic granulomatous disorder driven by immune dysregulation, often manifests with lymphadenopathy, organomegaly, and elevated inflammatory biomarkers, while CMML is a clonal myeloid neoplasm characterized by monocytic proliferation and cytopenias. We report a case of CMML-1 in a 48-year-old man with a 14-year history of sarcoidosis, presenting with progressive dyspnea, and pancytopenia. Laboratory tests revealed unusual elevations in key immune markers, prompting further investigation. This case highlights the diagnostic pitfalls in distinguishing between granulomatous disease and clonal hematologic malignancy in patients with longstanding sarcoidosis. It underscores the importance of maintaining a high index of suspicion for myeloid neoplasms when unexplained cytopenias occur and emphasizes the emerging diagnostic utility of biomarkers such as sIL-2R in differentiating chronic immune activation from underlying clonal hematopoiesis.

## Introduction

Chronic myelomonocytic leukemia (CMML) is a clonal hematopoietic stem cell disorder that exhibits overlapping features of myelodysplastic syndromes and myeloproliferative neoplasms. It is characterized by persistent monocytosis, varying degrees of cytopenias, dysplasia in one or more myeloid lineages, and often splenomegaly. The 2022 World Health Organization (WHO) classification introduced significant updates to the diagnosis and classification of CMML: CMML-1: <5% in peripheral blood and <10% in bone marrow; CMML-2: 6-19% in peripheral blood and 10-19% in bone marrow [1]. Despite updated criteria, CMML remains a diagnostic and therapeutic challenge due to its clinical heterogeneity and variable risk of progression.

Sarcoidosis is a multisystem granulomatous disease of unclear etiology, marked by the formation of non-caseating granulomas. It most commonly affects the lungs and intrathoracic lymph nodes, but may also involve the liver, spleen, skin, and eyes. The pathophysiology is driven by an exaggerated T-cell-mediated immune response to an unidentified antigen, resulting in granuloma formation and potential organ dysfunction. Classical findings include bilateral hilar lymphadenopathy on chest imaging, pulmonary symptoms such as dry cough and dyspnea, skin lesions like erythema nodosum and lupus pernio, ocular inflammation (uveitis), and the presence of non-caseating granulomas on tissue biopsy, which serves as the diagnostic hallmark. Biomarkers such as soluble interleukin-2 receptor (sIL-2R) and serum angiotensin-converting enzyme (sACE) levels are frequently used to monitor disease activity and extrapulmonary involvement in sarcoidosis [2,3]. However, the sIL-2R marker along with β2-microglobulin can also be elevated in hematologic malignancies such as CMML, complicating diagnosis when both conditions are present [4,5].

Although sarcoidosis has been linked to a variety of malignancies, including lymphomas (particularly Hodgkin’s lymphoma), myeloproliferative disorders, and solid tumors such as lung, breast, and skin cancers, the mechanisms behind this association remain unclear. Sarcoidosis may be seen as a paraneoplastic phenomenon, where the immune response to the tumor triggers granuloma formation, or it may be a result of shared genetic or environmental risk factors. The co-occurrence of sarcoidosis with hematologic cancers, including lymphomas and leukemia, is not uncommon; however, the coexistence of sarcoidosis with CMML is exceedingly rare [6-8]. We present a case of CMML-1 diagnosed in a patient with longstanding sarcoidosis and discuss the diagnostic challenges encountered in differentiating between these overlapping conditions.

## Case presentation

A 48-year-old male with a known history of sarcoidosis involving the liver and spleen, diagnosed 14 years earlier and treated with high-dose corticosteroids for more than a year, presented with progressive exertional dyspnea, persistent dry cough, low-grade fever, polyuria, and dysuria. These symptoms had developed insidiously over several months. He denied weight loss or night sweats. Past medical history was notable for Hashimoto’s thyroiditis, essential tremor, congenital hearing loss, and rubella encephalitis in childhood.

**Figure 1:**
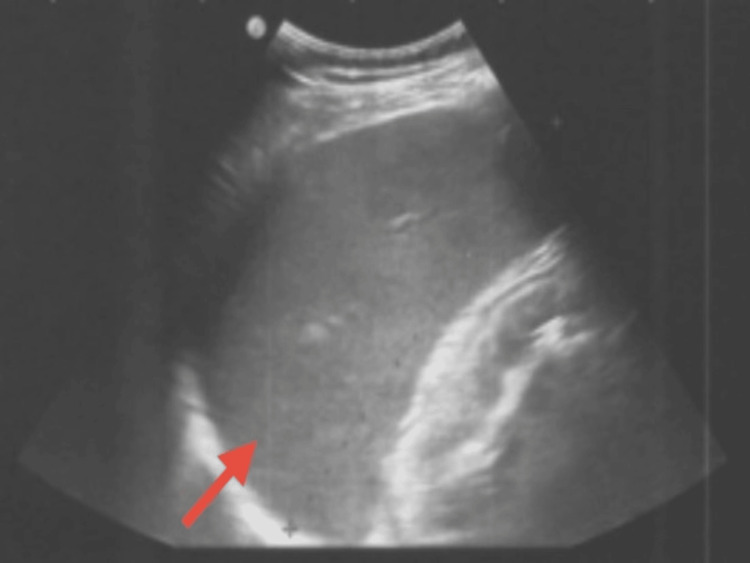
Abdominal ultrasound revealing marked splenomegaly measuring approximately 20 cm in length (red arrow).

On examination, he was euvolemic, afebrile, and had no signs of peripheral edema or cyanosis. Cardiac and pulmonary auscultation were normal. Abdominal examination showed no palpable organomegaly, though ultrasound revealed marked splenomegaly (20 cm) (Figure 1). A neurological evaluation confirmed a coarse tremor of the head and hands.

Laboratory investigations showed pancytopenia (hemoglobin (Hb) 11.0 g/dL, white blood cell (WBC) 2.95×10⁹/L, platelet count (Plt) 97×10⁹/L, hematocrit (Hct) 32%) with elevated monocyte levels (38%) and a blast count of 3%, elevated transaminases (aspartate aminotransferase (AST) 77 U/L, alanine transaminase (ALT) 61 U/L), hypercalcemia (serum calcium 2.62 mmol/L), and elevated D-dimer (13.62 µg/mL). Inflammatory and immunologic markers demonstrated significantly elevated sIL-2R (16,836 U/L), lactate dehydrogenase (LDH) (474 U/L), angiotensin-converting enzyme (ACE) (87 U/L), and β2-microglobulin (7.12 mg/L) (Table 1). Urine testing showed hypercalciuria (urinary calcium 10.4 mmol/24h). Doppler ultrasound of the lower extremities ruled out deep vein thrombosis.

**Table 1:** Key laboratory abnormalities at initial presentation. Reference ranges reflect the standards used at Christliches Krankenhaus Quakenbrück (CKQ), Quakenbrück, Germany. Hb = hemoglobin; WBC = white blood cells; Plt = platelet count; Hct = hematocrit; sIL-2R = soluble interleukin-2 receptor; sIL-2R = soluble interleukin-2 receptor; ACE = angiotensin-converting enzyme; LDH = lactate dehydrogenase; AST = aspartate aminotransferase; ALT = alanine transaminase

Laboratory Test	Result	Units	Reference Range	Interpretation
Hb	11.0	g/dL	13.5-17.5	Low
WBC Count	2.95	×10⁹/L	4.0-10.0	Low
Monocytes	38	%	1.0-14.0	High
Blasts	3	%	0%	Slightly elevated
Plt	97	×10⁹/L	150-400	Low
Hct	32	%	38-50%	Low
sIL-2R	16,836	U/mL	223-710	High
β2-Microglobulin	7.12	mg/L	0.8-2.2	High
ACE	87	U/L	8-52	High
LDH	474	U/L	140-280	High
AST	77	U/L	<50	High
ALT	61	U/L	<50	High
Calcium (Serum)	2.62	Mmol/L	2.15-2.55	High
D-Dimer	13.62	µg/mL	<0.5	High
Calcium (Urine)	10.4	Mmol/24h	2.5-7.5	High

**Figure 2:**
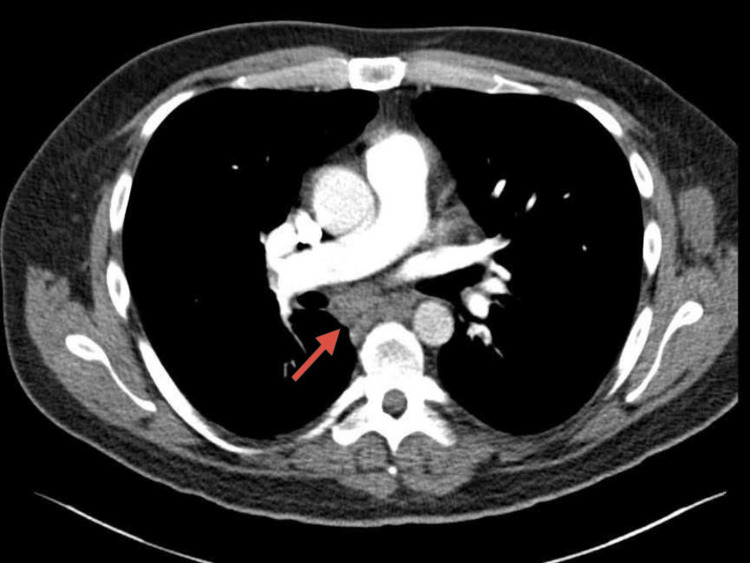
Axial chest CT scan showing pathologically enlarged mediastinal lymph nodes (red arrow), with the largest measuring approximately 2 cm in the left paratracheal region.

Contrast-enhanced thoracic CT angiography excluded pulmonary embolism but showed multiple enlarged lymph nodes in the mediastinum, hila, abdomen, and axillae, with a left axillary lymph node conglomerate measuring approximately 12 cm in cranio-caudal dimension (Figure 2). A small calcified granuloma and pleural scarring were noted in the right upper lobe. Fungal infections and tuberculosis were ruled out. Cardiac MRI showed preserved left ventricular function with no evidence of cardiac sarcoidosis. Brain MRI with venous angiography ruled out sinus vein thrombosis and neurosarcoidosis.

**Figure 3:**
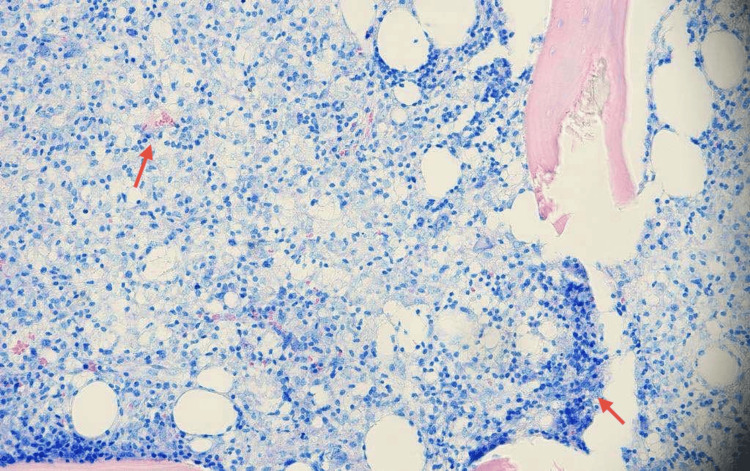
Bone marrow biopsy stained with Giemsa showing residual hematopoiesis and focal infiltration by histiocytic and monocytic cells (highlighted by red arrows), with preservation of overall marrow architecture. No granulomatous inflammation is observed (magnification ×20).

**Figure 4:**
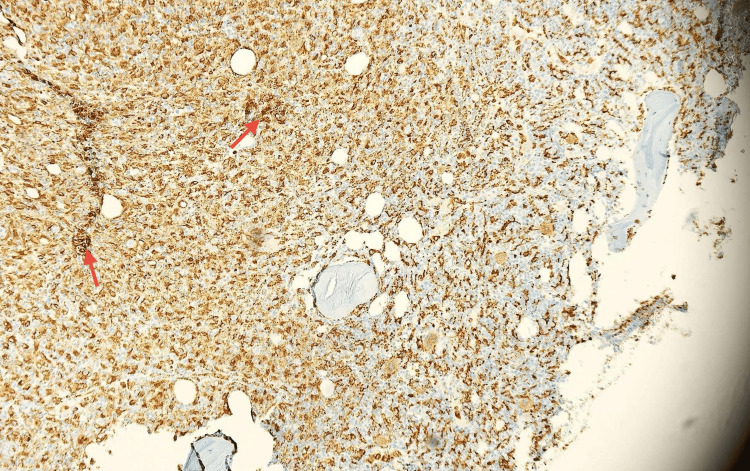
Immunohistochemical staining for CD68 highlighting widespread positivity in monocytes and histiocytic cells (highlighted by red arrows), confirming histiocytic proliferation within the bone marrow (magnification ×20).

Flow cytometry shows no evidence of an atypical B- or T-cell subpopulation. A small myeloid blast population was identified in the CD45⁻/SSC image, expressing CD34, CD117, CD13, CD33, and HLA-DR, along with a mature monocyte population. After permeabilization, no monoclonal plasma cell population is detected. Given the persistence of unexplained pancytopenia, a bone marrow biopsy was performed (Figure 3; Figure 4).

Histopathologic examination showed diffuse proliferation of monocytes (4.5%) with a blast count of 6%, histiocytic cells, and T-lymphocytes with increased expression of CD68 and CD3. No granulomatous inflammation was identified. Population of CD34+/CD117+ myeloid blasts (<10%) and polytypic plasma cells were also observed. Erythropoiesis is reduced, with maturation proceeding in a normoblastic pattern (CD71-positive). There is a reduction in megakaryocyte number, with evidence of displacement from typical marrow niches (CD61-positive), findings that are suggestive of megakaryocytic dysplasia. These findings were highly suggestive of a myelodysplastic/myeloproliferative neoplasm, specifically CMML-1. Subsequent bone marrow flow cytometry confirmed a small blast population expressing CD34, CD117, CD13, CD33, and HLA-DR. Blasts comprised less than 10% of nucleated bone marrow cells, consistent with a diagnosis of CMML-1 (Table 2). 

**Table 2:** Diagnostic criteria of CMML (WHO 2022). CMML = chronic myelomonocytic leukemia; WBC = white blood cells; CML = chronic myeloid leukemia; MPNs = myeloproliferative neoplasms; WHO = World Health Organization

Category	Criteria
Prerequisite Criteria	1. Absolute monocyte count ≥0.5×10⁹/L and ≥10% of total WBC in peripheral blood.
	2. Blasts: <20% in peripheral blood and bone marrow.
	3. Exclusion: Not meeting criteria for CML, other MPNs, or myeloid/lymphoid neoplasms with eosinophilia and defining gene rearrangements (e.g., PDGFRA, PDGFRB, FGFR1, JAK2).
Supporting Criteria	1. Dysplasia: In ≥1 myeloid lineage.
	2. Clonality: Acquired clonal cytogenetic or molecular abnormality (e.g., mutations in TET2, SRSF2, ASXL1, SETBP1).
	3. Monocyte Subsets: Abnormal partitioning of peripheral blood monocyte subsets.
Diagnostic Requirements	- Prerequisite criteria must be present in all cases.
	- If monocytosis ≥1.0×10⁹/L: One or more supporting criteria must be met.
	- If monocytosis <1.0×10⁹/L: Supporting criteria 1 and 2 must be met.
Blast Subgroups	- CMML-1: <5% blasts in peripheral blood and <10% in bone marrow.
	- CMML-2: 6-19% blasts in peripheral blood and 10-19% in bone marrow.

## Discussion

The co-occurrence of sarcoidosis and CMML presents a rare but important diagnostic challenge. Serratrice et al. reported two cases of sarcoidosis preceding CMML, suggesting a potential link between the two conditions [8]. The intersection of granulomatous inflammation and clonal myeloid neoplasia raises questions about shared immunopathogenic mechanisms and diagnostic pitfalls. Both diseases involve dysregulation of the monocyte/macrophage lineage and aberrant T-cell activity [9,10]. Recent studies have highlighted how the inflammatory processes in sarcoidosis, particularly the accumulation of activated T-cells and macrophages, may create an environment that predisposes individuals to clonal hematopoiesis and the development of CMML. Both sarcoidosis and CMML are associated with an altered immune response, including increased sIL-2R levels, suggesting that similar immune dysregulation may underlie both conditions. Expanding our understanding of these shared mechanisms is crucial for improving the diagnosis and treatment of patients presenting with both sarcoidosis and CMML [11,12].

The overlap in clinical features, such as lymphadenopathy, splenomegaly, elevated inflammatory markers, and systemic symptoms, can obscure diagnosis. Both sarcoidosis and CMML can present with these findings [2,10]. In our case, markedly elevated sIL-2R and β2-microglobulin initially suggested sarcoidosis reactivation, as elevated sIL-2R levels have been observed in sarcoidosis patients [5], and β2-microglobulin is a known marker of tumor burden in myeloid malignancies [3]. However, the absence of granulomas on bone marrow histology and the presence of monocytic proliferation and CD34+/CD117+ blasts pointed toward CMML. A comparison of key biomarkers further highlights the diagnostic overlap between the two conditions (Table 3).

**Table 3:** Comparison of key biomarkers in sarcoidosis and CMML, and their diagnostic significance. Biomarker data adapted from [2] and [10]. CMML = chronic myelomonocytic leukemia; sIL-2R = soluble interleukin-2 receptor; ACE = angiotensin-converting enzyme; LDH = lactate dehydrogenase

Biomarker	Typical in Sarcoidosis	Reported in CMML	Notes	Reference Range
sIL-2R	Frequently elevated	May be elevated	Non-specific; reflects immune activation	223-710 U/mL
β2-microglobulin	May be mildly elevated	Often elevated	Marker of tumor burden in CMML	0.8-2.2 mg/L
ACE	Often elevated	Not typical	Specific but variable sensitivity in sarcoidosis	8-52 U/L
LDH	Mildly elevated	Often elevated	Suggests cell turnover or marrow infiltration	140-280 U/L

Sarcoidosis has long been associated with hematologic malignancies, particularly lymphomas, a relationship known as sarcoidosis-lymphoma syndrome. However, associations with myeloid neoplasms like CMML are rare and underrecognized. Serratrice et al. [8] reported two cases of sarcoidosis preceding CMML, both of which involved cutaneous and ocular manifestations, and they proposed that the shared involvement of the monocyte/macrophage lineage might suggest a spectrum of immune dysregulation, progressing from non-clonal granulomatous inflammation to clonal hematologic neoplasia. Our patient similarly demonstrated longstanding immune activation, characterized by elevated sIL-2R levels and chronic granulomatous inflammation, before developing CMML.

Recent molecular studies have identified frequent mutations in genes such as TET2, SRSF2, ASXL1, and SETBP1 among patients with CMML [13,14]. These findings support the hypothesis that chronic inflammatory environments, such as those seen in sarcoidosis, may promote somatic mutations and clonal evolution [15]. Autoimmune phenomena, including vasculitis, polyarthritis, and immune cytopenias, are observed in up to 30% of CMML patients [16]. Moreover, a Danish registry study demonstrated that autoimmune conditions significantly increase the risk of developing CMML [17], suggesting that chronic inflammation may drive clonal hematopoietic evolution [18].

Furthermore, the use of immunosuppressive therapy in sarcoidosis, such as corticosteroids, may create an immunologic environment favoring oncogenesis [7], although our patient had been off steroids for several years prior to his CMML diagnosis. Interestingly, HTLV-1 proviral DNA has been detected in sarcoid lesions but not in peripheral blood, raising the possibility of localized viral triggers contributing to immune dysregulation [19].

Other reported overlaps between sarcoidosis and hematologic malignancies include acute myelomonocytic leukemia and leukemia cutis [20]. Additionally, there are rare reports of simultaneous resolution of both sarcoidosis and leukemia following donor lymphocyte infusion after bone marrow transplantation [19], further supporting the hypothesis of a shared immune-mediated mechanism.

Although CMML-1 represents the least aggressive subtype, it still carries a risk of progression to acute myeloid leukemia. Prognosis depends heavily on cytogenetic abnormalities and specific gene mutations, particularly TET2, SRSF2, and ASXL1, which are associated with adverse outcomes [13].

Our case provides additional clinical support for the hypothesis that chronic granulomatous inflammation, such as longstanding sarcoidosis, may foster a pro-inflammatory milieu conducive to clonal hematopoietic evolution and the development of CMML [15,17]. Greater recognition of this association may prompt earlier hematologic evaluation and intervention in sarcoidosis patients presenting with unexplained cytopenias.

## Conclusions

This case underscores the rare and diagnostically challenging co-occurrence of sarcoidosis and CMML, highlighting the importance of considering clonal hematologic disorders in sarcoidosis patients who develop new-onset cytopenias or persistent systemic symptoms. The overlapping clinical and biomarker features between these conditions, particularly elevated sIL-2R and β2-microglobulin levels, can obscure the diagnosis and delay appropriate treatment. Our findings emphasize the value of a multidisciplinary, biomarker-guided diagnostic approach and raise important questions about the immunologic interplay between chronic inflammation and myeloid neoplasia. Greater recognition of this association may facilitate earlier diagnosis, timely hematologic referral, and improved patient outcomes through molecular risk stratification.

## References

[1] (2025). Chronic myelomonocytic leukemia (CMML). https://www.mll.com/en/myelodysplastic-myeloproliferative-neoplasm-mds-mpn/chronic-myelomonocytic-leukemia-cmml.

[2] Kraaijvanger R, Janssen Bonás M, Vorselaars AD, Veltkamp M (2020). Biomarkers in the diagnosis and prognosis of sarcoidosis: current use and future prospects. Front Immunol.

[3] Grutters JC, Fellrath JM, Mulder L, Janssen R, van den Bosch JM, van Velzen-Blad H (2003). Serum soluble interleukin-2 receptor measurement in patients with sarcoidosis: a clinical evaluation. Chest.

[4] Yokose N, Ogata K (1997). Plasma soluble interleukin-2 receptors in patients with myelodysplastic syndromes. Leuk Lymphoma.

[5] Norfolk DR, Child JA, Roberts BE, Forbes MA, Cooper EH (1983). Serum beta-2-microglobulin in disorders of myeloid proliferation. Acta Haematol.

[6] Patt YS, Ben-Shabat N, Sharif K (2024). The association between sarcoidosis and malignancy: a comprehensive population-based cohort study. J Clin Med.

[7] Voutidou S, Eleftheriadis D, Drakopanagiotakis F, Papanikolaou IC, Steiropoulos P (2025). Pathogenetic mechanisms linking sarcoidosis to lymphoma. Int J Mol Sci.

[8] Serratrice J, Granel B, Swiader L (2002). Sarcoidosis preceding chronic myelomonocytic leukemia. Report of two cases. Dermatology.

[9] Zhang H, Costabel U, Dai H (2021). The role of diverse immune cells in sarcoidosis. Front Immunol.

[10] Patnaik MM, Tefferi A (2020). Chronic myelomonocytic leukemia: 2020 update on diagnosis, risk stratification and management. Am J Hematol.

[11] Fraser SD, Crooks MG, Kaye PM, Hart SP (2021). Distinct immune regulatory receptor profiles linked to altered monocyte subsets in sarcoidosis. ERJ Open Res.

[12] Addinsell HM, Cant R, Hull NJ, Wang YH, Somervaille TC, Wiseman DH, Batta K (2025). Multi-omic analysis of chronic myelomonocytic leukemia monocytes reveals metabolic and immune dysregulation leading to altered macrophage polarization. Leukemia.

[13] Rossille D, Gressier M, Damotte D (2014). High level of soluble programmed cell death ligand 1 in blood impacts overall survival in aggressive diffuse large B-Cell lymphoma: results from a French multicenter clinical trial. Leukemia.

[14] Itzykson R, Kosmider O, Renneville A (2013). Clonal architecture of chronic myelomonocytic leukemias. Blood.

[15] Avagyan S, Zon LI (2023). Clonal hematopoiesis and inflammation - the perpetual cycle. Trends Cell Biol.

[16] Patnaik MM, Tefferi A (2022). Chronic myelomonocytic leukemia: 2022 update on diagnosis, risk stratification, and management. Am J Hematol.

[17] Elbæk MV, Sørensen AL, Hasselbalch HC (2016). Chronic inflammation and autoimmunity as risk factors for the development of chronic myelomonocytic leukemia?. Leuk Lymphoma.

[18] Pietras EM (2017). Inflammation: a key regulator of hematopoietic stem cell fate in health and disease. Blood.

[19] Tauro S, Mahendra P (2001). Resolution of sarcoidosis after allogeneic bone marrow transplantation with donor lymphocyte infusions. Bone Marrow Transplant.

[20] Rodríguez-Rivera DV, Cuesta-Camunas J, Ramos-Rodriguez AJ (2022). A case of aleukemic leukemia cutis mistaken for sarcoidosis. J Am Acad Dermatol.

